# Microbiota-Immune Interaction in the Pathogenesis of Gut-Derived Infection

**DOI:** 10.3389/fimmu.2019.01873

**Published:** 2019-08-07

**Authors:** Chenyang Wang, Qiurong Li, Jianan Ren

**Affiliations:** Research Institute of General Surgery, Jinling Hospital, Medical School, Nanjing University, Nanjing, China

**Keywords:** gut-derived infection, gut microbiota, mucosal immunity, intestinal epithelium, bacterial translocation, intestinal barrier

## Abstract

Gut-derived infection is among the most common complications in patients who underwent severe trauma, serious burn, major surgery, hemorrhagic shock or severe acute pancreatitis (SAP). It could cause sepsis and multiple organ dysfunction syndrome (MODS), which are regarded as a leading cause of mortality in these cases. Gut-derived infection is commonly caused by pathological translocation of intestinal bacteria or endotoxins, resulting from the dysfunction of the gut barrier. In the last decades, the studies regarding to the pathogenesis of gut-derived infection mainly focused on the breakdown of intestinal epithelial tight junction and increased permeability. Limited information is available on the roles of intestinal microbial barrier in the development of gut-derived infection. Recently, advances of next-generation DNA sequencing techniques and its utilization has revolutionized the gut microecology, leading to novel views into the composition of the intestinal microbiota and its connections with multiple diseases. Here, we reviewed the recent progress in the research field of intestinal barrier disruption and gut-derived infection, mainly through the perspectives of the dysbiosis of intestinal microbiota and its interaction with intestinal mucosal immune cells. This review presents novel insights into how the gut microbiota collaborates with mucosal immune cells to involve the development of pathological bacterial translocation. The data might have important implication to better understand the mechanism underlying pathological bacterial translocation, contributing us to develop new strategies for prevention and treatment of gut-derived sepsis.

## Introduction

Bacterial infections are common complications in critically ill patients, likely leading to sepsis, multiple organ dysfunction, and even death (1). Infection and septic complications contributed to the majority of deaths in these cases, and are regarded as the leading cause for mortality in critical illness (2, 3). Elucidation of the mechanisms underlying the pathogenesis of infection and septic complications in critical illness is therefore of upmost importance, facilitating to develop potentially effective strategies for prevention and treatment.

In critical illness, the gut may serve as the motor of multiple organ dysfunction syndromes (MODS), probably derived from intestinal bacterial translocation and subsequent acute septic responses (4, 5). Early in this decade, studies regarding bacterial translocation mainly focused on the structure and function of intestinal epithelial barrier (6). Disruption of the epithelial barrier and increased gut permeability have been frequently observed in critically ill patients, which was thought playing a central role in the development of bacterial translocation and systemic infections in these cases (7, 8). The gut microbiota has long been recognized as a key component of the intestinal barriers (9). It has been known that small intestinal bacterial overgrowth could pre-dispose to bacterial translocation (10, 11), however, there have been few efforts to characterize the composition and dynamic changes of the gut microbiota in the process, due to technological limitations. Over the past 15 years, the introduction of next-generation DNA sequencing techniques has revolutionized this area of science, allowing us to define the microbial compositions and their potential functions in the intestine (12). Recently, the gut microbiotas in critically ill patients have been determined through high-throughput sequencing analyses, characterized by overgrowth of pathogenic organisms and the loss of commensal bacteria (13–16). The gut microbiota dysbiosis could contribute to bacterial translocation by increasing gut permeability and inducing the mucosal immune dysfunction (17). The findings demonstrate that the microbiota is probably an active participant in the development of gut-derived infection, sepsis, and multiple-organ dysfunction in critical illness (18, 19). Thereby, improved knowledge of the gut microbiota composition and function would facilitate more comprehensive understanding of the mechanisms behind the pathogenesis of gut-derived infection in critical illness and the design of new treatment options.

The gut microbiota serves as a critical player in preventing and sometimes in driving enteric infections (20). Trillions of commensal microorganisms residing in the gastrointestinal (GI) tract can compete for adhesion sites with pathogens, and comprise the first line of defense against bacterial translocation (21). Alterations in the intestinal microbiota induced by antibiotics treatment can lead to the translocation of enteric bacteria across the epithelium in mice (22), providing further evidence for the importance of the microbiota in host resistance against pathogens. In addition to this, the gut microbiota has a key role in maintaining the gut homeostasis by establishing and maintaining beneficial interaction with mucosal immune cells and intestinal epithelial cells (23). In critical illness, this interaction could become pathological due to alterations of the gut microbiota, leading to the loss of intestinal homeostasis, bacterial translocation, gut-derived sepsis, and deleterious clinical sequelaes (24). Thereby, it is needed to unravel the changes of the gut microbiota and the underlying mechanisms of microbiota–host interaction in critical illness, contributing to offer new strategies to reconstruct intestinal homeostasis and avoid some of the untoward outcomes.

Based on the current research data, gut microbiota perturbations, host immune deficiencies, and increased intestinal permeability are the three key factors responsible for promoting bacterial translocation and gut-derived infection. Given the crucial role of the microbiota in shaping intestinal barrier integrity, it is interesting to consider whether microbiota dysbiosis and altered microbiota–host interaction is causally linked to gut-derived infection and consequent septic complications. In this review, we presented the changing features of the intestinal microbiota structure and composition in critical illness and the potential roles of these changes in the pathogenesis of gut-derived infection. We also discussed how the gut microbiota drives bacterial translocation through alterations in microbial community architecture, modulation of innate and adaptive immunity, and disruption of the mucosal barrier in critical illness. The data presenting here have highlighted the alterations of the microbiota–immune interaction in critical illness and offer novel paradigms to understand the pathophysiology of gut-derived sepsis. We also reviewed the research advances on other components (fungi, parasites, and viruses) of the gut microbiota and their potential relationships with bacteria and host immunity in human health and diseases. Lastly, we discussed the therapeutic potential to modify the intestinal microbiota with fecal microbiota transplantation (FMT).

## Bacterial Translocation and Gut-Derived Infection

Bacterial translocation is defined as the process in which the intestinal bacteria and/or their products spread through the gut barrier into the extra-intestinal sites, including the mesenteric lymph nodes (MLNs), systemic circulation, and distant organs (25, 26). The phenomenon of bacterial translocation was initially described in 1949, when live enteric bacteria were observed in the peritoneal washings from dogs with hemorrhagic shock (27). Until 1990s, however, the translocation of enteric organisms into the mesenteric lymph node (MLN) was identified in surgical patients undergoing laparotomy (28–30), which offered direct evidence supporting this concept. Bacterial translocation was also associated with a striking increase in the post-operative sepsis, leading to the generation of the gut origin hypothesis of sepsis. Subsequently, a large amount of clinical studies further confirmed the presence of bacterial translocation in patients with critical illness and its involvement in the development of sepsis (31–34). Based on the findings, it began to be accepted that bacterial translocation is a major source of systemic infections and might play an important early step in the pathogenesis of sepsis in critically ill patients (35, 36).

In the last several decades, detection of bacterial translocation in patients is mainly dependent upon culture of peripheral blood (37). Owing to low sensitivity of this method, cultures of blood specimens are often negative, even in the patients with sepsis (38). As a result, specific interventions against infections are probably delayed in some cases, causing lethal complications. It is quite possible that enteric bacteria may translocate into systemic circulation, but escape from detection by culture-based methods. In recent years, the development of 16S rDNA-based molecular techniques has improved the ability to detect the microorganisms, allowing us to define the composition of translocating bacteria into the blood (39, 40). Using denaturing gradient gel electrophoresis, multiple organisms (5–8 bacterial species) were frequently observed in the blood specimens of severe acute pancreatitis (SAP) patients (41). Recent studies with next-generation sequencing techniques showed that a diverse microbiota is present in the blood of septic patients and is mainly composed of gut-associated microorganisms (42–44), indicating the possibility for translocation of intestinal microbiota. Dickson et al. demonstrated that the lung microbiome is enriched with gut-associated bacteria both in a murine model of sepsis and in patients with acute respiratory distress syndrome (ARDS) (45). Furthermore, the lower GI tract, rather than the upper respiratory tract, was identified as the likely source community of post-sepsis lung microbiota, providing evidence for gut–lung translocation of intestinal microbiota (45, 46). Based on culture-dependent methods, previous studies have demonstrated that the bacterial translocation is usually characterized by migration of one or several organisms from the gut (29, 30). Discovery and identification of the blood and lung microbiota has prompted us to rethink the notion of bacterial translocation, which might be replaced by translocation of gut microbiota. Although many observations have strongly supported the hypothesis of the gut microbiota translocation, future studies with next-generation sequencing techniques are needed to characterize the microbial landscape in the MLN and distant organs in patients and experimental models. The findings would provide direct evidence for the translocation of intestinal microbiota and give us new perspectives to understand the pathogenesis of gut-derived sepsis. Interestingly, recent studies have revealed that in healthy individuals the blood and lung also harbor a diverse bacterial microbiota (44, 45, 47, 48), suggesting that translocation of intestinal microbiota may present under healthy condition. The observations are consistent with previous opinion that intestinal bacterial translocation probably occurs as a normal physiological event in healthy subjects (49). However, the pathological translocation of enteric bacteria in critically ill patients may increase owing to breakdown of intestinal barrier integrity (50), likely causing the alterations in the blood and lung microbiotas and the pathogenesis of systemic infections and sepsis (44, 45).

## Dysbiosis of Intestinal Microbiota and Gut-Derived Infection

In the past few decades, our understanding into the structure and function of the gut microbiota has been largely enriched with advances of culture-independent techniques. The gut microbiota is involved in maintaining host homeostasis, with an important role in nutrition and energy metabolism (51), immune modulation (52), and host defense (53). Recently, numerous studies have highlighted the composition and role of the gut microbiota under a range of intestinal and extraintestinal diseases (54–62). The involvement and implication of the gut microbiota in the development of bacterial translocation and gut-derived infection have also been broadly recognized. The harmful roles that the intestinal microbiota plays in critical illness are multifactorial and may be separated into three aspects: disruption of microbial barrier, loss of colonization resistance and metabolic disorder (63–65).

### Disruption of Microbial Barrier and Gut-Derived Infection

The gut microbiota represents the first barrier of protection against pathogen invasion, and disruption of this barrier is probably required for gut-derived infection in critical illness. Recent data showed that the intestinal microbiotas in critically ill patients in intensive care unit (ICU) are significantly altered, as characterized by overgrowth of opportunistic Proteobacteria and decreases in commensals Firmicutes and Bacteroidetes (13–16). Of special note, the presence of specific pathogens at ICU admission was associated with subsequent infection with the same organism for *Escherichia coli, Pseudomonas* spp., *Klebsiella* spp., *Clostridium difficile*, and vancomycin-resistant *Enterococcus* (66). Furthermore, *Enterococcus* status at ICU admission was associated with risk for death or all-cause infection, indicating that the gut microbiota alterations have potential impact on mortality or the risk of healthcare-associated infections in critically ill patients (67). The patients with SAP also had significant alterations in the gut microbiota, including reduced microbiota diversity, increased *Enterococcus* and *Enterobacteriaceae*, and decreased *Bifidobacterium* (68). Additionally, the changes of the gut microbiota have been frequently seen in patients who underwent severe trauma (69), serious burn (70, 71), and major surgery (72, 73). The dysbiosis of the microbiota has been linked to occurrence of severely adverse events in critical illness, including sepsis, MODS, and even death (74, 75). Of special note, altered microbiota composition could cause increased penetrability and a deteriorated colonic mucus layer, contributing to lethal colitis and susceptibility to infection by enteric pathogens, such as *C. difficile* (76) and *Citrobacter rodentium* (77). Apparently, this is becoming clearer that the gut microbiota seems to provide disease-promoting influences in critically ill patients. A plethora of data from basic research with animal models also supports the prominent role of the gut microbiota dysbiosis in contributing to adverse outcomes in critical illness (78, 79). For instance, intestinal ischemia/reperfusion (I/R) injury could trigger a dysbiosis of gut microbiota and mucosal barrier damage, leading to enteric bacterial translocation and development of septic complications (80, 81). Altogether, the microbiota dysbiosis in critical illness is among the key factors that cause dysfunction of the intestinal barrier, contributing to pathological bacterial translocation and gut-derived infection. Yet, the extent to which this dysbiosis is causative to the subsequent acute septic response and multiple organ failures observed in critical illness remains to be determined.

### Decreased Colonization Resistance Against Intestinal Pathogens

The intestinal microbiota plays a critical role in resistance against colonization by exogenous bacterial pathogens, termed colonization resistance (82). This phenomenon has been described over 50 year ago, and it has long been thought as microorganism-mediated direct inhibition (83). Being present in such huge numbers, the microorganisms in intestinal tract can compete for limited nutrition and adherence sites to the epithelia, preventing overgrowth, and invasion of potentially pathogenic microbes. Long-term antibiotic treatment could cause loss of commensal enteric bacteria, and thus decreases this direct inhibition. As a result, antibiotic-resistant bacterial species, such as vancomycin-resistant *Enterococcus faecium* (63), Gram-negative *Enterobacteriaceae* (84), and *C. difficile* (85), could proliferate and dominate mucosal surfaces, preceding severely enteric infection and bloodstream invasion. In addition to its direct roles in nutrition and niche competition, the gut microbiota can also combat invading pathogens indirectly by enhancing host immune defenses (immune-mediated colonization resistance) in the gut. The commensal bacteria are capable of augmenting mucosal immune responses for eradication of invading pathogens by various mechanisms (86–88). Overall, both direct and indirect mechanisms could cooperate to provide resistance against colonization and invasion by potential pathogens, preventing the occurrence of bacterial translocation and gut-derived infection. Although the mechanisms underlying colonization resistance remain incompletely defined, there is little doubt that reestablishing colonization resistance after antibiotic treatment could be a potentially effective strategy for prevention and therapy of antibiotic-resistant bacterial infection. Recent studies have proved that the commensal microbiotas can be successfully manipulated to cure *C. difficile* infection in patients (89), which has been regarded as a consequence of reestablishing microbiota-mediated colonization resistance.

### Potential Role of Microbial Metabolic Disorders

The gut microbiota has a huge metabolic activity and can convert host-derived and dietary components (lipids, carbohydrates, proteins, etc.) into various metabolites that are either beneficial or harmful for the host (90). Some of the metabolic products, including lactic acid, short chain fatty acids, bile salts, and bacteriocins are often considered as antimicrobial factors playing a critical role in protection against pathogenic infection (91, 92). On the contrary, a few metabolites deriving from microbial digestion of proteins, such as phenolic and sulfur-containing compounds, are potentially toxic to intestinal epithelial cells (93). The phenol expose could cause an increase of paracellular permeability in a dose-dependent manner, due to destruction of the intercellular tight junctions (94, 95). Likely, the microbiota alterations in critically ill patients might induce metabolic disorders and excessive production of such toxic metabolites, resulting in disruption of intestinal epithelial barrier and bacterial translocation (96, 97).

In total, increasing evidence has demonstrated that the microbiota dysbiosis is closely associated with the development of gut-derived sepsis and subsequent mortality in critically ill patients (19, 98). As such, the gut microbiota has also been successfully used as a therapeutic target in the management of sepsis and MODS (99–101). With emerging evidence from clinical trials and basic researches, the causality of the relationship between the microbiota dysbiosis and gut-derived sepsis would be demonstrated. It will raise hope for simple and effective adjunctive therapies based on our expanding knowledge of the gut microbiota that might benefit critically ill patients.

## Microbiota-Immune Interaction and Gut-Derived Infection

The intestinal immune system is considered as the last but the most important defense line against invasion of enteric microorganisms. There is a dynamic and complex interaction between the gut microbiota and the mucosal immune system (102). Under normal conditions, the microbiota could maintain a delicate balance with the mucosal immune system, which is extremely important for host health (54). The critical illness and associated medical interventions can cause a rapid and extreme change in the gut microbiota composition and activation of mucosal immune response (103). Consequently, this interaction between the gut microbiota and mucosal immune system is strikingly altered and becomes pathological in nature, providing the possibility for bacterial translocation, gut-derived infection and deleterious clinical sequalae.

### Communication Between Gut Microbiota and Innate Immunity

In order to confront the microbial challenges, the intestine has developed a complex immune defense network containing the greatest number and diversity of immune cells in the body. As an important component of the intestinal immune network, the innate immune system plays a pivotal role in maintaining the balance between tolerance to commensal microorganisms and immunity to opportunistic pathogens (104). The innate immune cells in the intestine are usually non-responsive to the great number of commensal microorganisms. Yet, they can sense enteric microbial signals to restrict overgrowth of the pathobionts and assure a beneficial microbiota composition. At the same time, the innate immune cells also can rapidly respond to invading pathogens and prevent migration from the intestinal lumen to systemic circulation and distant organs. Once passing the mucous and epithelial barriers, invading bacteria would be recognized, phagocytosed, and eliminated by mucosal innate immune cells (e.g., macrophages, dendritic cells) under healthy state (105). Since the critically ill patients are usually accompanied by systemic immune deficiencies or immunosuppression, the innate immune cells in intestinal mucosa are likely dysfunctional and fail to eradicate invading pathogens, and thus lead to systemic translocation of intestinal bacteria (106–108). Translocating bacteria and their products can activate immune response through recognition of specific pathogen-associated molecular patterns (PAMPs) by host innate immune cells (e.g., neutrophils and macrophages), triggering a systemic inflammatory response (109). Under such pathological conditions, activated neutrophils are excessively recruited into the intestine, which further promotes a dysregulation of innate immune function and cause mucosal injury (110). Alterations of the enteric microenvironment, coupled with medical treatment, lead to an overgrowth in opportunistic pathogenic bacteria and a decrease of commensal bacteria in critical illness (13–16). The dysbiotic microbiota, in turn, could aggravate the mucosal immune dysfunction and promote an increase in enteric bacterial translocation, ultimately resulting in gut-derived infection, sepsis, and MODS (111, 112). Unsurprisingly, the interaction between gut microbiota and mucosal innate immunity is severely perturbed during the process. The innate immune dysregulation, microbiota dysbiosis, and bacterial translocation seem to shape a positive-feedback loop, together leading to uncontrollable inflammatory response and septic complications in critical illness. In a mouse model, morphine treatment induced a shift of gut microbiota toward a proinflammatory phenotype, which may be a result of the innate immune changes and commensal bacterial translocation (113–115). Yet, fecal microbiota transplant successfully reversed morphine-induced microbial dysbiosis and restored gut immune homeostasis (113). The findings provide evidence supporting the existence of the feedback loop and its potential importance in the pathogenesis of gut-derived infection.

Several antimicrobial molecules generating from goblet cells, Paneth cells, and enterocytes, also have been identified as critical components of the innate immunity (116). These substances, including mucins, defensins, lysozyme, secretory phospholipase A2, and cathelicidins, have strong microbicidal activity and are able to directly kill microbes in the intestine, facilitating maintenance of gut homeostasis (117, 118). The generation and release of such antimicrobial molecules is also regulated by the gut microbes and their products (119, 120). Owing to lack of gut microbial stimulations, the intestinal mucous layer in germ-free mice is remarkably attenuated, despite the numbers of goblet cells are normal (121). Introduction of bacterial products, such as lipopolysaccharide (LPS) or peptidoglycan, can stimulate the release of mucin by goblet cells, leading to a rapid reconstitution of the inner mucous layer (122). The metabolites of the gut microbiota, i.e., butyrate, also can promote release of mucin for maintenance of the mucous barrier (123). The antimicrobials from Paneth cells, including defensins, lysozyme, and secretory phospholipase A2, are also expressed under the control of gut microorganisms (124). In return, the antimicrobial functions of these substances are required for stabilization of the gut microbiota (125) and integrity of the epithelial barrier (116, 126). In mice deficient for principal intestinal mucin (Muc2), there is an increased translocation of commensal and pathogenic bacteria (127), which is closely related to bacterial overgrowth in the intestine. In cynomolgus monkeys, administration of Campath-1H, a humanized monoclonal antibody against CD52, led to a significant decrease in the expression of defensin 5 and lysozyme in Paneth cells, altering the composition of the gut microbiota toward a pathogenic state (128). Likewise, it has been reported that decreased expression of α-defensins due to loss of Paneth cells can induce an expansion of pathogenic bacteria and a reduction in gut microbial diversity, leading to bacterial translocation (129). In addition, a lack of the antimicrobial cathelecidin can cause more severe disruption of intestinal mucosa in the colitis mouse models induced by dextran sodium sulfate (130). Evidently, diminished release of antimicrobial molecules is involved in increased bacterial translocation and is, at least in part, responsible for the pathogenesis of gut-derived infection.

In addition to bacterial translocation, one of the most interesting aspects regarding gut microbiota and host innate immunity involves *C. difficile* infection (CDI) and *C. difficile*-associated diarrhea (CDAD) (131). Many studies have indicated that the composition and diversity of the fecal microbiota in patients with CDI are pronouncedly altered, and the dysbiosis is associated with the infection and its resistance to antibiotic therapy (132, 133). A variety of factors, including antibiotics, NSAIDs, acid suppressing agents, and ages, can cause the microbiota dysbiosis. The loss of the protective microbial barrier allows for the formation of an ecological niche that favors the growth of *C. difficile*, and then leads to CDI and CDAD. Several mechanisms, such as alterations of fermentative metabolism (especially SCFAs), alterations of bile acid metabolism, and imbalance of antimicrobial substances production, have been proposed to explain the involvement of the microbiota in the process of the infection (131). Unsurprising, the innate immune system also participates in the pathogenesis of CDI, which is mainly mediated via toxin-dependent mechanism (134). Following colonization and growth of *C. difficile* in the intestinal tract, the innate immune cells (135, 136), including intestinal mast cells, macrophages, monocytes, and dendritic cells, are activated by *C. difficile* toxins, through the surface and intracellular innate immune sensors, for instance, the inflammasome and the TLR4, TLR5, and NOD1 signaling pathways (137). Multiple proinflammatory cytokines (IL-12, IL-18, IFN-γ, IL-1β, TNF-α) and chemokines (MIP-1a, MIP-2, IL-8, leptin) are produced in the process, which may be responsible for host inflammatory damages and the histopathological features associated with CDI, such as fluid accumulation, edema, increased mucosal permeability, mast cell degranulation, epithelial cell death, and intense local neutrophilic infiltration (138). Collectively, the microbiota dysbiosis and impaired innate immune response could play crucial roles in triggering *C. difficile* colonization and growth, and in the development of CDAD.

### Crosstalk Between Gut Microbiota and Mucosal Adaptive Immunity

Despite characterized by tolerance to enteric microorganisms, the intestinal immune system has the daunting task of protecting us from pathogenic insults. Apart from the innate immunity, a highly sophisticated adaptive immune system also has been evolved in the gut (139), which are of upmost importance for prevention of bacterial translocation and gut-derived infection. When the enteric microorganisms cross the epithelium, the adaptive immune cells in the intestine are activated by antigen-presenting cells (macrophages, dendritic cells) to eradicate pathogens and establish long-lasting protective immunity (140). In the intestine, there is a huge and diverse population of T lymphocytes, forming a large part of the adaptive immune response. Many studies have suggested that loss of mucosal T cells has significant adverse effects on the maintenance of intestinal barrier integrity and defense of enteric infection, leading to increased morbidity (141, 142). In burn-injured rats, translocation of intestinal bacteria to MLN and systemic circulation is markedly increased following depletion of T cells (143). Gut I/R can induce a significant reduction in T-cell numbers and variations in lymphocyte phenotypes in intestinal mucosa, leading to enteric bacterial translocation and development of septic complications (144, 145). Depletion of intestinal mucosal lymphocytes induced by Campath-1H could cause dysbiosis of gut microbiota (128, 146, 147) and disruption of intestinal epithelial barriers (148, 149). Similar to the observations, severe impairment of gut barrier integrity was also seen in intestinal transplanted patients receiving Campath-1H administration (150, 151), which might be a major reason for high incidence of infectious complications after small bowel transplantation. In both septic patients and animal sepsis models, the lymphocytes within the intestinal epithelium undergo significant apoptosis, leading to pathologic bacterial translocation and gut-derived sepsis (152–165).

The adaptive immune system in the gut mucosa is mainly composed of intraepithelial lymphocytes (IELs) and lamina propria lymphocytes (LPLs) (156). They are essential to the adaptive immune response in intestinal mucosa, and have been shown to play a critical role in defending against the invasion of pathogens and infections. When the adaptive immune system is disrupted, the translocation of intestine-derived bacteria occurs and could trigger systemic inflammatory response and the onset of sepsis. γδ T cells are a unique subset of T cells with a distinct T-cell receptor (TCR), and serve as a key controller for the adaptive immune response to a broad range of pathogens (157). Intraepithelial γδ T lymphocytes can prevent mucosal dissemination of bacteria through the secretion of cytokines and antimicrobial molecules following mucosal injury (158). In the absence of intraepithelial γδ T cells, the host control of invasive bacteria is compromised and invasive bacteria populations are expanded (159). Additionally, the reduction of γδ T cells in the gut mucosa could induce transition of non-invasive intestinal bacterial types toward more invasive, causing bacterial translocation into the systemic circulation and pathological infections. In septic patients, γδ T cells in peripheral blood are significantly reduced, and this decrease is closely associated with the high mortality rate caused by infectious complications (160, 161).

The gut microbiota is actively involved in shaping and maintaining normal adaptive immune system in intestinal mucosa (139). The phenotypic differentiations of specific lymphocyte lineages in the mucosal immune system are reliant on the distinct component of the microbiota. In germ-free mice, the gut adaptive immune system is underdeveloped, and introduction of the commensal bacteria can induce enrichment and differentiations of mucosal lymphocytes (162–164). Development of the adaptive immune cell diversifications represents an establishment of a complete “firewall” in the gut, which could prevent against the translocation of indigenous bacteria and pathogen infection (165). The gut microbiota also plays an important role in modulating the production of secretory IgA, mainly targeting against the enteric commensals and their antigens (166, 167). In the absence of IgA, the gut commensal bacteria could more easily enter the lamina propria and submucosal tissue by leaky barrier, leading to enteric bacterial translocation (168–170). The individuals with secretory IgA deficiency have a tendency to develop gut-derived infections and functional disorders of the intestinal tract (171, 172). The interaction between gut microbiota and mucosal immunity is extremely complex. Consequently, the precise mechanism by which the alteration in commensal bacteria-specific adaptive immunity crosstalk involves the invasion and translocation of enteric bacteria remains incompletely clear and needs to be further elucidated.

## Other Organisms Beyond Bacteria in the Intestinal Tract

In addition to the bacteria, the human intestinal microbiota also contains fungi, viruses, parasites, and other organisms. Despite representing a smaller fraction of the gut microbiota, they also play a crucial role in maintaining host health and in driving the development of the intestinal diseases.

### Gut Fungal Microbiota

In GI tract, the fungi comprise a dynamic and ecologically diverse microbial community, termed the gut mycome. The fungal microbiota has been regarded as a critical player for the development of fungal infections and intestinal diseases, through interacting with enteric bacteria and host immune system (173). In ICU patients, the fungal overgrowth in the gut is frequently presented, which is usually considered as a result of commensal enteric bacteria loss after antibiotic or immunosuppressive therapy (174). Subsequently, the fungal pathogens, such as *Candida* and *Aspergillus*, could translocate impaired intestinal barrier into the bloodstream, leading to the fungemia. In a non-human primate model with lymphocyte depletion, the gut fungal microbiota is also perturbed, together with a dysbiosis of the bacterial flora (147). The findings indicate that a complex crosstalk may exist between the fungal and bacterial microbiota in the gut. It has been shown that *Candida albicans* has an ability to modify the bacterial microbiota (175), however, the detailed mechanisms underlying this interaction are still not well-known. There is also a complex interaction between the fungal microbiota and host immune system, which is mainly mediated via an innate immune receptor Dectin-1 (176). After recognizing β-1,3-glucans (a component of the fungal cell walls), Dectin-1 could activate intracellular signals through CARD9, resulting in release of inflammatory cytokines and induction of Th17-mediated immune responses (176, 177). Deficiencies in either Dectin-1 or CARD9 can lead to enhanced susceptibility to pathogenic fungal infections in humans and mice (178, 179), and are closely associated with ulcerative colitis in humans (180, 181). With improved understanding into host-fungus relationships, several fungal species with beneficial effects have been utilized in many acute and chronic diseases. For example, *Saccharomyces boulardii* has showed significant efficacy in preventing antibiotic associated diarrhea (182) and relapse of *C. difficile* infection (183). Despite these advances, in-depth studies on gut mycome composition and their relationships with gut bacteria, host immunity and related diseases are still warranted.

### Intestinal Parasites

The intestinal parasites, mainly including Blastocystis and Amoebozoa, represent a unique microeukaryotic population, also termed gut eukaryome. Over the past few decades, the advances of DNA-based molecular techniques have enabled us to better estimate the presence of the intestinal parasites and its roles playing in human health and gastrointestinal diseases (184). Recent studies with real-time PCR showed that single-celled parasites, such as Blastocystis and Dientamoeba, are far more common than previously anticipated, even in developed countries (185, 186). Intriguingly, these parasites are most common in individuals with a healthy gut, while less prevalent in patients with irritable bowel syndrome (IBS) (187), and even less common in patients with inflammatory bowel disease (IBD) (188). The observations suggest that the parasites may be beneficial to human health rather than culprits of diseases (189). However, the parasites infection is possibly present in some individuals, which may be associated with specific ecological conditions in the gut, such as the microbiota dysbiosis. Gilchrist et al. showed that a high parasite burden was coupled with increased abundance of *Prevotella copri* in Bangladeshi children with *Entamoeba histolytica* infection (190). In a mouse model with n amoebic colitis, the microbiota dysbiosis induced by antibiotic treatment can increase the severity of amoebic colitis and delay the clearance of *E. histolytica* (191). *Giardia* infection was also related to the dysbiosis of gut microbiota, as characterized by an increase of facultatively and strictly aerobic bacteria (192). In contrast to this, some animal experiments showed that probiotics can prevent or modulate parasite infection, supporting the association of the gut microbiota with the parasites (193). Taking all these studies into account, it appears that the presence of intestinal parasites, are closely linked to certain microbial communities. However, the causative link between the presence of a given parasite and the microbiota dysbiosis is still incompletely clear. The gut microbiota may not only be driving the susceptibility to, but also the outcome of, parasite infection (194). Future investigations should be designed to strengthen our knowledge regarding associations between parasites and gut microbiota, and also explore whether the parasites can be transplanted to a diseased recipient as a potential therapy for functional and/or organic bowel diseases as well as metabolic disorders.

### Gut Virome

The human gut virome is composed of two main players: microbial viruses (bacteriophages) and eukaryotic viruses (195). It is estimated that the human GI tract contains ~10^15^ bacteriophages, which represent the most abundant member of the gut virome (196). The vast majority of bacteriophages in the gut are a DNA phage named crAssphage (cross-assembly phage), mainly belonging to the family Podoviridae (197). Similar to the bacterial microbiome, the gut viral communities are established at birth and evolve over time to become “adult-like” virome (198, 199). The structure and composition of the virome are also influenced by age, host genetics and environmental factors, such as diet, antibiotic use, and location (198–202). The viruses also have cross-kingdom interaction with the bacteria and other constituents of the intestinal microbiota, which are usually beneficial to host health and sometimes could increase the risk of disease (203). Owing to their ability to kill host bacteria, the phages can play a role in maintenance of the intestinal homeostasis through affecting the structure and function of enteric bacterial community (204). Under certain conditions, however, changes of the phage populations could induce intestinal dysbiosis and contribute directly to the development of intestinal diseases, such as IBD (205). To explain the mechanisms underlying phage-driven intestinal dysbiosis, several hypothetical models (206), including “Kill the Winner” model, “Biological Weapon” model, and “Community Shuffling” model, have been put forward to elucidate the complex interaction between the phages and bacteria during the process. In addition to these, the phages can also transfer genes (i.e., bacteriophage transcription factors) into bacteria to change their phenotypes and further control their biological functions, which is termed as the “Emerging New Bacterial Strain” model. Meanwhile, enteric bacteria also develop defense mechanisms against the bacteriophages, through the restriction modification system (207), hiding membrane receptors (208), increasing production of competitive inhibitors (209), self-destruction (210), and CRISPR-Cas systems (211). The detailed mechanisms that maintain the balance between bacteriophages and bacterial populations and result in the intestinal dysbiosis and diseased states have been documented in the review article by Mukhopadhya et al. (212). Development and implementation of metagenomic techniques have allowed us to study the “entire virome” composition and its interaction with other elements of the gut microbiome. With discovery and identification of new viral genomic sequences in the coming years, our understanding on the gut virome as a cohesive ecological unit that can affect the intestinal homeostasis and lead to diseases will continue to improve.

## Manipulation of Gut Microbiota for Treatment of Gut-Derived Sepsis

Considering the gut microbiota dysbiosis as one of the most important factors that can lead to pathologically bacterial translocation and systemic infection, it may be feasible to develop novel therapeutic strategies against gut-derived sepsis by modulating the microbiota. More than 90% of the commensal organisms would be lost during the early stage of the critical illness insults, thereby, it may be impossible that a single or several probiotic species would be able to completely replenish the diversity of the gut microbiota (213). Transfer of healthy donor feces containing thousands of microbial species, termed FMT, would facilitate replenishment of diminished commensal bacteria and guide the patient's microbiota toward a healthy state (214). In the last several years, FMT has been successfully utilized in the treatment of recurrent CDI (215, 216). Yet, FMT is scarcely used in the treatment of septic patients, due to that in such cases antibiotic therapy is frequent and its continuation would adversely influence remodeling of the microbiota after FMT. Recently, it has been reported on the use of FMT in septic patients with MODS and non-*C. difficile* diarrhea, refractory to standard medical management (99–101). At 2–3 weeks of post-FMT, the patients had resolution in their diarrhea and significant decreases in the blood levels of the inflammatory mediators, such as TNF-α, interleukin (IL)-1β, IL-6, and C-reactive protein. Following FMT, the stool microbiotas in the patients showed marked alterations toward that of the donors, with growing Firmicutes and reducing Proteobacteria. Even though this is a serial of case reports, the improved clinical outcomes in these patients following FMT are still exciting. This success raises the possibility for the use of the unconventional therapeutic procedure in the clinical management of gut-derived sepsis and MODS which is commonly complicated in critically ill patients. Although the efficacy of FMT observed in such cases reports remains to be further validated, manipulation of the microbiota with FMT for therapeutic benefits represents a new avenue in the future care of critically ill patients (16, 75, 217–219). Nonetheless, such early experiences with FMT curing ICU patients have strengthened enthusiasm for broader its use in critical illness.

## Concluding Remarks

The interplay between gut microbiota and host immune is exquisitely complex. Exploration of the relationship between the gut microbiota alterations and host immunological disorders has significant potential to enhance our understanding and future treatment of relevant diseases. Abundant evidence has demonstrated that disturbance of the microbiota-immune relationship is a key event in the development of pathological bacterial translocation (220, 221). However, studies of the microbiota-immune interaction in critical illness remain in their infancy, and the underlying mechanisms are still incompletely clear. Beyond just describing effects of the microbiota dysbiosis on mucosal immune cell phenotypes, future investigations need to move toward unraveling the molecular mechanisms of the interaction in the pathogenesis of gut-derived infection. Systems biology studies based gut metagenomics and immunogenomics under the conditions of critical illness have fundamental importance for identifying the critical signal pathways and molecules that promote translocation of enteric microorganisms. Elucidation of the cross-regulation of gene expression between commensal bacteria and cells of the mucosal immune system will provide us mechanistic understanding on the complex interaction in critical illness. The knowledge would enable the field to enter a stage in which interventional strategies could be designed to improve the immune defense against invading microorganisms while protecting from pathological bacterial translocation to systemic circulation. With deeper understanding of this interaction, the precision manipulations that can restrict bacterial translocation may be possible and offer new strategies to avoid some of the untoward outcomes related to gut-derived infection in critically ill patients.

## Author Contributions

CW wrote the original draft and revised the manuscript. QL reviewed and edited the manuscript. JR critically revised the manuscript. All authors read and approved the final version of the manuscript for submission.

### Conflict of Interest Statement

The authors declare that the research was conducted in the absence of any commercial or financial relationships that could be construed as a potential conflict of interest.
